# Micropatterning of biologically derived surfaces with functional clay nanotubes

**DOI:** 10.1080/14686996.2024.2327276

**Published:** 2024-03-25

**Authors:** Mingxian Liu, Rawil Fakhrullin, Anna Stavitskaya, Vladimir Vinokurov, Nisha Lama, Yuri Lvov

**Affiliations:** a Department of Materials Science and Engineering, Jinan University, Guangzhou, P. R. China; b Institute of Fundamental Medicine and Biology, Kazan Federal University, Kazan, Russian Federation; c Department of Physical and Colloid Chemistry, Gubkin Russian State University of Oil and Gas, Moscow, Russian Federation; d Institute for Micromanufacturing, Louisiana Tech University, Ruston, LA, USA

**Keywords:** Clay nanotubes, nanocomposites, functional biomaterials, halloysite

## Abstract

Micropatterning of biological surfaces performed via assembly of nano-blocks is an efficient design method for functional materials with complex organic–inorganic architecture. Halloysite clay nanotubes with high aspect ratios and empty lumens have attracted widespread interest for aligned biocompatible composite production. Here, we give our vision of advances in interfacial self-assembly techniques for these natural nanotubes. Highly ordered micropatterns of halloysite, such as coffee rings, regular strips, and concentric circles, can be obtained through high-temperature evaporation-induced self-assembly in a confined space and shear-force brush-induced orientation. Assembly of these clay nanotubes on biological surfaces, including the coating of human or animal hair, wool, and cotton, was generalized with the indication of common features. Halloysite-coated microfibers promise new approaches in cotton and hair dyeing, medical hemostasis, and flame-retardant tissue applications. An interfacial halloysite assembly on oil microdroplets (Pickering emulsion) and its core–shell structure (functionalization with quantum dots) was described in comparison with microfiber nanoclay coatings. In addition to being abundantly available in nature, halloysite is also biosafe, which makes its spontaneous surface micropatterning prospective for high-performance materials, and it is a promising technique with potential for an industrial scale-up.

## IMPACT STATEMENT

This international group of authors unites researchers who pioneered halloysite clay nanotubes for biomaterials, and discloses a new strategy for this nanoclay composite design through interfacial architecture. These results confirm Dr. K. Ariga concept of nanoarchitectonics, and demonstrate promising applications. Assembly of the clay nanotubes on biosurfaces, including the coating of human or animal hair, wool, and cotton, was generalized for the process optimization. Halloysite-coated microfibers promise new approaches in cotton and hair dyeing, and medical hemostasis and flame-retardant tissue applications. Related techniques of interfacial halloysite assembly on oil microdroplets (Pickering emulsion) and its quantum dots core–shell structure for cell imaging are also described. Contrary to many other synthetic nanomaterials, described natural halloysite nanotubes are environmentally safe and abundantly available, thus allowing for scale up of the suggested functional biocomposites.

## GRAPHICAL ABSTRACT

**Figure:**
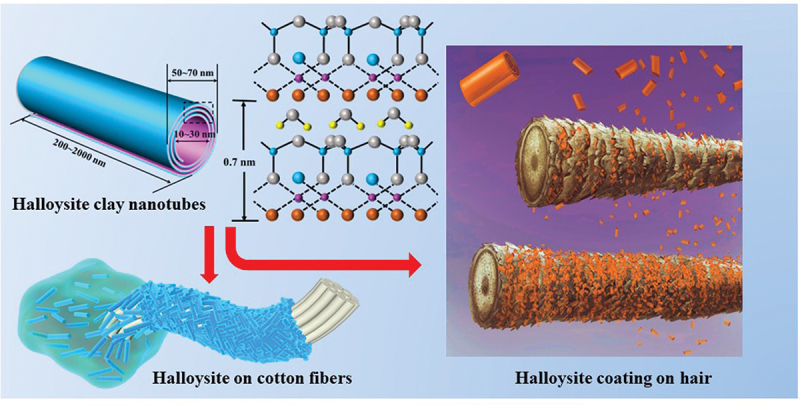

## 1. Halloysite clay nanotubes – characterization, modification, and loading

Nanoarchitectural approach in functional materials design is a promising strategy for building up at integrated scales, combining nano and micro features in macroscopic devices. In this approach, one manipulates with nanoscale blocks (macromolecules or atom clusters), assembling them in the predetermined order. Dr. Katsuhiko Ariga is one of the world leaders in nanoarchitectonics, which exploits different interactions, such as an interfacial assembly of amphiphilic molecules (Langmuir–Blodgett films), electrostatic attraction of charged molecular ensembles (layer-by-layer coating and encapsulation), and in other systems, often containing carbon composites, gold coating with thiol bound organic shells or bimetallic nanoparticles [1,2]. One can observe examples of nanoarchitectonics design in materials with tunable properties for medicine, microbiology, photonics, electronics, meso-catalysis, and smart chemical composites. In this paper, we discuss architectural prospects of natural clay nanotubes, as a part of this designing strategy.

**Scheme 1.**
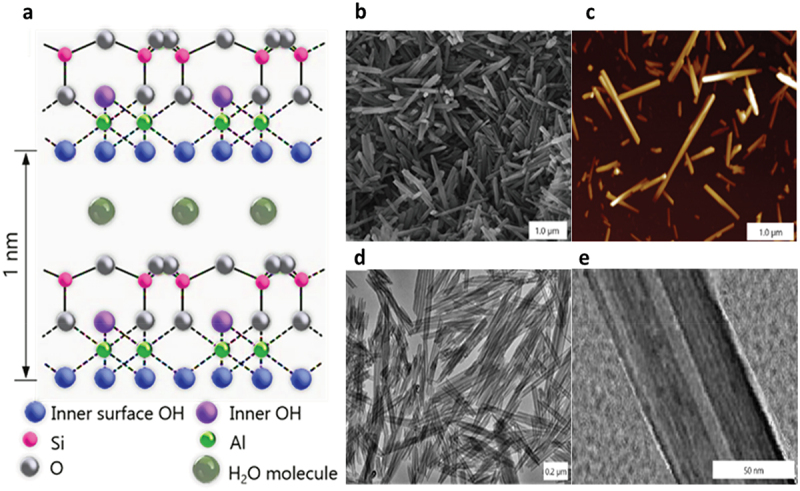
Halloysite structure (a), and scanning electron microscopy (SEM) (b), atomic force microscopy (AFM) (c), and transmission electron microscopy (TEM) images of the nanotubes. Reproduced with permission from [7], copyright by Wiley, 2016.

Halloysite nanotubes (HNT) are naturally occurring tubule form of kaolin clay with a diameter of about 50–70 nm, an empty internal lumen of 10–15 nm, and a length of 0.6–0.8 μm (larger diameter and longer halloysite tubes are rare, as well as none-tubular halloysite). It is a natural and biocompatible nanomaterial available in thousands of tons at 90–95% purity from selected pockets in kaolin deposits. Halloysite has a density of 2.53 g/cm^3^, a cationic exchange capacity of 30–50 CMol/kg, and surface area of ca 60 m^2^/g. Halloysite is formed by rolling 20–25 kaolin sheets under favorable geological conditions. In HNT structure, SiO_2_ sits on the outside, making it overall negatively charged, and Al_2_O_3_ is on the inside of the nanotubes, forming its lumen that is positively charged at pH range 4–8. HNT chemical formula is Al_2_Si_2_O_5_(OH)_4_ • nH_2_O, where *n* = 2, 4. In dehydrated samples *n* = 2, with the interwall spacing being 0.72 nm and for cases of swelled row halloysite, *n* = 4 with 1.0 nm of wall spacing, Scheme 1. An electric zeta-potential (ζ) of HNT is at a threshold of colloidal stability of ca −30 mV at pH 4–8.5, and this ζ-potential may be roughly explained by an algebraic summation of the outside layer of silica with potential of ca −50 mV and the inside alumina layer with ζ-potential of ca +20 mV [3–7]. This provides its colloidal stability in water for 2–3 h at concentrations of a few percent after which precipitation of larger tubes may be noticed. Contrary to kaolin, bentonite, and other platy clays, halloysite nanotube clusters may be easily dispersed by a short sonication or stirring in water. Halloysite undergoes a phase transition at ca 490°C when hydroxyl groups are removed, while preserving 90 wt % of its mass and the tubular shape. HNT has a low water contact angle of about 20 ± 5°, indicating its hydrophilicity.

The properties of HNT make it a promising carrier for numerous compounds including functional chemical agents, drugs, proteins, DNA, and RNA. An opposite inner and outer charge of the tubes allows for the selective inner loading of negative compounds in the positively charged lumen, and at the outside surface – for adsorption of positive compounds [3,7,8]. Acid etching of alumina can help to enlarge halloysite inner lumen diameter and porosity to maximize the loading ability to 30%. Taking the tube sizes into account, one may estimate a limit of inner loading as 10–12 wt % for pristine, unetched HNT, while on the outer surface, it may be immobilized with about twice as many positive compounds. A basic method for HNT inner loading is mixing of the nanoclay powder with 4–5 times amount of chemical solutions at the highest (close to saturated) concentrations. A capillary pressure of water in HNT lumen is estimated as ca 200 atm, which should be sufficient for enforcing materials inside the tubes. However, as a standard procedure, three-time cyclic air pumping (out and in) is used, assuming that this procedure removes air trapped inside the lumens, replacing it with the solution [3].

Polar solvents such as water, alcohol, and acetone were most convenient. Loading HNT with anticorrosion, antibacterial, and flame-retardant compounds and mixing them to paint or polymers at 4–5 wt % allowed for fabrication of functional organic–inorganic composites with long-lasting protection properties [6]. Loading drugs into HNT lumen at 9–10 wt % allowed for formulations with sustained 20–50-h release, efficient for topical medical applications (HNT is already used in cosmetics) [9]. However, HNT-drug formulation for blood injections is questionable because this nanoclay is not biodegradable, though it is safe for external medications or as a tablet component.

One of the promising aspects of HNT is the possibility of protein loading from water solutions. It is possible to reach 6–8 wt % loading inside the lumens for negative proteins and 10–15 wt % for cationic proteins on the outer HNT surface (protein charge may be varied by selecting a pH below or above its isoelectric point). About half of the loaded proteins were released within the first 20 h, while the remaining fraction of tighter bound proteins was released within the next 20–30 h. HNT-loaded enzymes (such as glucose oxidase, urease, or peroxidase) showed higher thermal stability along with enhanced biocatalytic propensity at pH ranging above the optimum one [10]. Another important case is DNA immobilization with HNT. It is easily achievable by stirring compounds together, but the results are under discussion. These super large negative macromolecules may be in part located outside the clay nanotubes, increasing the complex zeta-potential negativity, and stabilizing the dispersion. In our opinion, this very long polyanions may partially anchor inside the positive HNT lumen and also wind around the nanotubes. Selective loading of enzymes and DNA into/onto these clay nanotubes is an important component of their architectural design.

There are two other important loading methods pioneered by Gua and Lazzaro groups [4,11]. They used silane compounds (e.g. octadecyl trimethoxysilane – ODTMS) to covalently bind amines to HNT outer surface with further chemicals reacting to this group. Another very interesting approach is based on 2–3 wt % adsorption of anionic amphiphiles such as sodium alcanoates (NaC_10_, NaC_12_, NaC_14_) selectively inside of positively charged lumen, thus converting it to a hydrophobic channel loosely covered with aliphatic chains [6]. Such lumens’ modification allowed for loading with decane and water-insoluble drugs and dyes from organic solvents. These results in a kind of inorganic tubule micelles that form stable aqueous colloids, which can be used to collect hydrocarbons or water-insoluble chemical agents.

Loading of HNT with any anionic compounds, for example, such as sodium polystyrene sulfonate or polyalginic acid, neutralizes the nanotubes’ internal positivity and partially extends outside, increasing the magnitude of HNT ζ-potential to ca −60 mV. Such a high electric potential allows for very stable HNT colloids in water (that can last several months) along with strong nanotubes interaction, resulting in aggregate phenomena, such as liquid crystallinity and orientation, promising for surface patterning [12].

## 2. Halloysite nanotube orientation in cell capturing patterns

**Figure 1.**
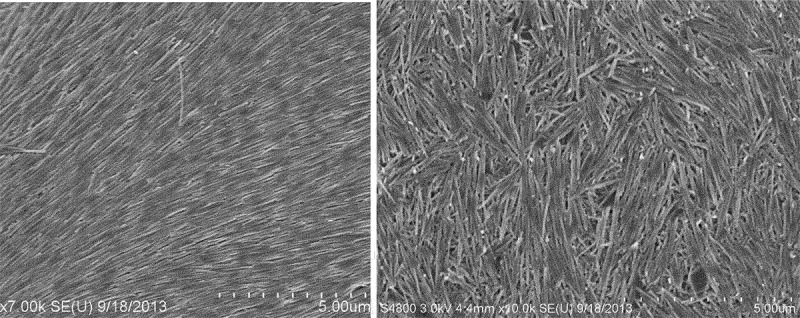
An edge of dried enhanced charge HNT-PSS droplet with nanotubes oriented, and non-oriented less charged pristine HNT (a, b).

**Scheme 2.**
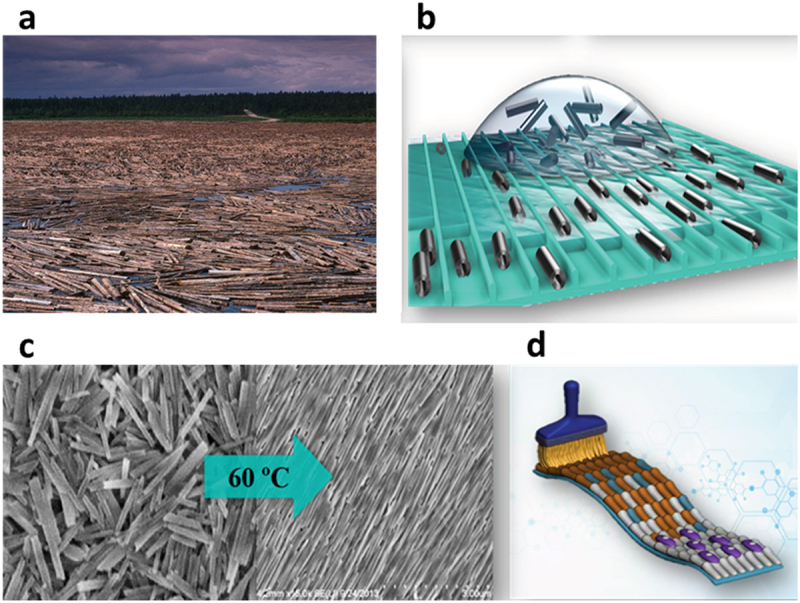
A photograph of wood logs aggregation in a river stream and halloysite nanotube orientation in microchannels (a, b), halloysite randomly oriented and aligned at the dried droplet edge (c), and brush ordering of clay nanotubes (d). Scheme 2d reproduced with permission from [17], copyright by Wiley 2019.

Halloysite clay nanotubes dispersed in water at concentrations of 5–20 wt % form lyotropic liquid crystalline regions, where these elongated particles (with a length-to-diameter anisotropic factor of ca 15) are oriented, so that these areas under crossed-polarizers microscopy appear to be colored nematic phase domains [13]. At larger concentrations, up to 35 wt %, liquid crystallite ordering spread over larger areas, eventually converting to the gel phase. These results inspired a search for methods to create ordered nanotube arrays for biomedical applications. We assume that there may be two mechanisms for HNT ordering: a steric interaction of elongated nanotubes and electrostatic repulsion due to a negative charge, which may also be enhanced. This allowed for three HNT orientation methods: electrostatically at the edge of drying droplets, as shown in Figure 1, or on plate dipped in the nanoclay dispersion, with shear-force by brash deposition or in microchannels or capillary flow, like electrospinning of HNT mixed with polymer solution [14–17], as illustrated in Scheme 2.

### 2.1. Halloysite in coffee rings, microchannels, and brush orientation

Following this strategy, we developed the electrostatic orientation of halloysite nanotubes at the edge of drying nanoclay aqueous droplets [14]. To enhance the HNT electric repulsion inducing orientation during drying, we increased the magnitude of its ζ-potential to −70 mV by loading anionic polystyrene sodium sulphonate (PSS) inside. HNT-PSS nanotubes form very stable aqueous colloidal dispersions. Such dispersion at a concentration of 0.2–0.5 wt % was dropped onto a hot support at 60–70ºC to accelerate water evaporation, forming dry rings of highly oriented halloysite.

**Figure 2.**
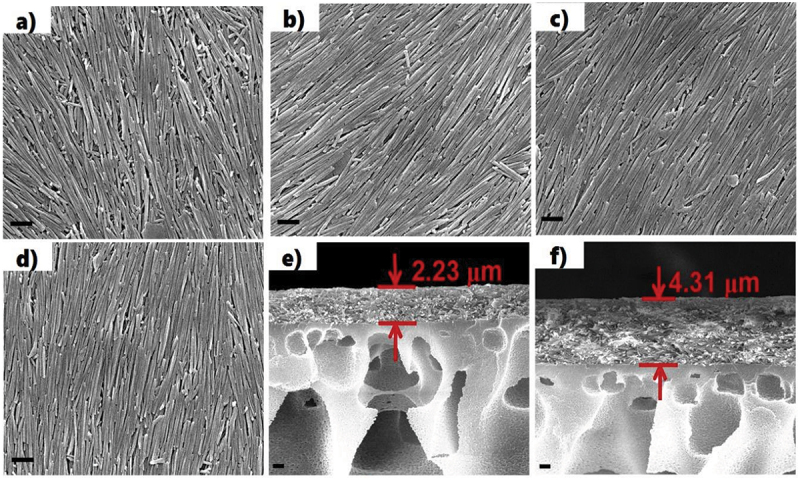
SEM upper images of polymeric membrane coated with oriented halloysite and cross-section for HNT-PSS-10 mg/mL (a, b, and e), HNT-PSS-20 mg/mL (c, d, and f) coating showing correlation of the nanoclay concentration and its layer thickness. Reproduced with permission from [18], copyrights by American Chemical Society, 2016.

A notable observation is the formation of a large-area, oriented halloysite coating, where a hot glass plate is vertically moving up through the water–air interface of the nanoclay dispersion, and a liquid crystalline membrane is formed on it. During evaporation of the solvent, horizontally oriented HNT domains are formed, as the aligned orientation consumes the least energy [15]. Practical applications of oriented clay nanotubes were demonstrated for composite nano/microporous filters (see Figure 2) and for electrically modulated liquid-crystalline HNT shields, allowing switch on/off of window transparency [18,19].

### 2.2. Orientation and capillary patterning for cell directions

Evaporation-induced self-assembly of droplets containing colloidal particles is an effective method for depositing nanoparticles with diversified resulting patterns [20]. Although the simple coffee-ring pattern is among the most common ones, its non-uniformity limits applications. Larger area orientation may be enhanced using brush deposition. Conveniently, the shearing force with brush HNT deposition aligns nanotubes on solid substrates [17]. HNTs were regularly aligned by brush shear force into strip-like patterns, accomplished with drying at elevated temperatures, with the best results observed at the clay concentration of 10–20 wt % (Scheme 2(d)).

The nanotubes’ orientation was governed by the ‘coffee-ring’ effect, which depends on the HNTs concentration, surface charge, brush type, drying speed, and substrate property. The nanotubes are aligned along the direction of the wetting lines at the dispersion concentration above 4 wt%, while they are not oriented at lower concentrations. The addition of sodium polystyrene sulfonate and surfactant sodium hexametaphosphate to HNT dispersion increases the surface charge (ζ-potential) and promotes uniform dispersion, which is beneficial to the alignment. It was found that nylon, cosmetic, and toothbrushes provide different levels of HNTs orientation in the patterns. The toothbrush has hard bristles that provide a significant shear force, presenting brighter orientation strips and color in polarized microscopy [17].

**Scheme 3.**
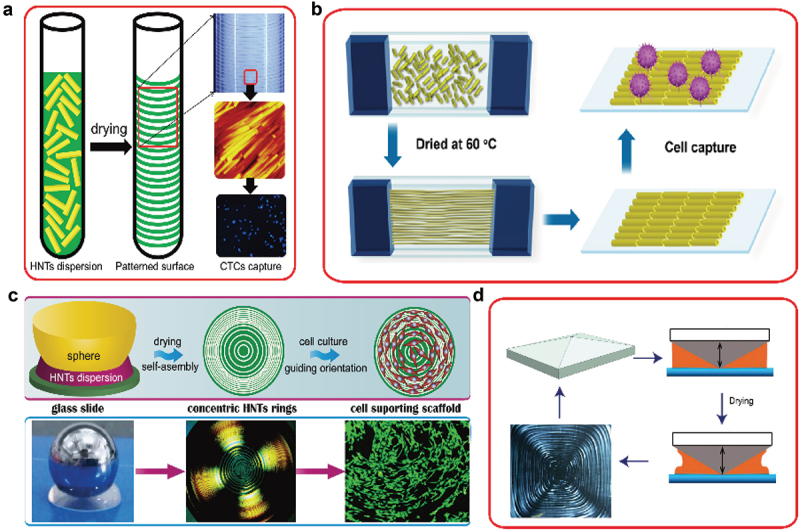
An assembly of HNTs on different substrates by heat-drying in confined space (a-capillary, where CTCs stands for circulating tumor cells, b- between vertical glass plates, c- concentric rings, and d– with a cone plate). Reproduced with permission from [21,25], copyright by American Chemistry Society 2016, 2017 and [24] Royal Society of Chemistry, 2017.

Simple but spectacular results were obtained by drying a capillary filled with HNT-PSS dispersion in an oven (Scheme 3). After a few hours of drying, the capillary inner wall was covered with rings of oriented nanotubes with a periodicity of ca 0.1 mm, which could be varied depending on the clay concentration [21]. The aligned HNTs patterns on different substrates containing glass, ceramic, tissue culture plate, and polymer scaffold provide a comprehensive platform for cell guidance. Human foreskin fibroblasts grew well on the aligned HNT patterns, and the cell orientation agreed with the nanotube direction. Human bone mesenchymal stem cells were also cultured on the organized HNT coating. The clay patterns supported stem cell proliferation with alignment, and such nanostructured substrates promoted osteogenesis differentiation without growth factors. The method has been extended with agitation-assisted assembly, in which the HNTs formed in ‘tree-ring’ micropatterns. The factors including nanotube charges, concentrations, drying temperature, droplet volume, and rotor size influenced the periodicity in concentric nanoclay circles [22]. These methods for preparing aligned clay nanotube patterns on solid substrates are promising for surface modification in bio-tissue engineering.

### 2.3. Capture of tumor cell by the nanotube pattern

**Figure 3.**
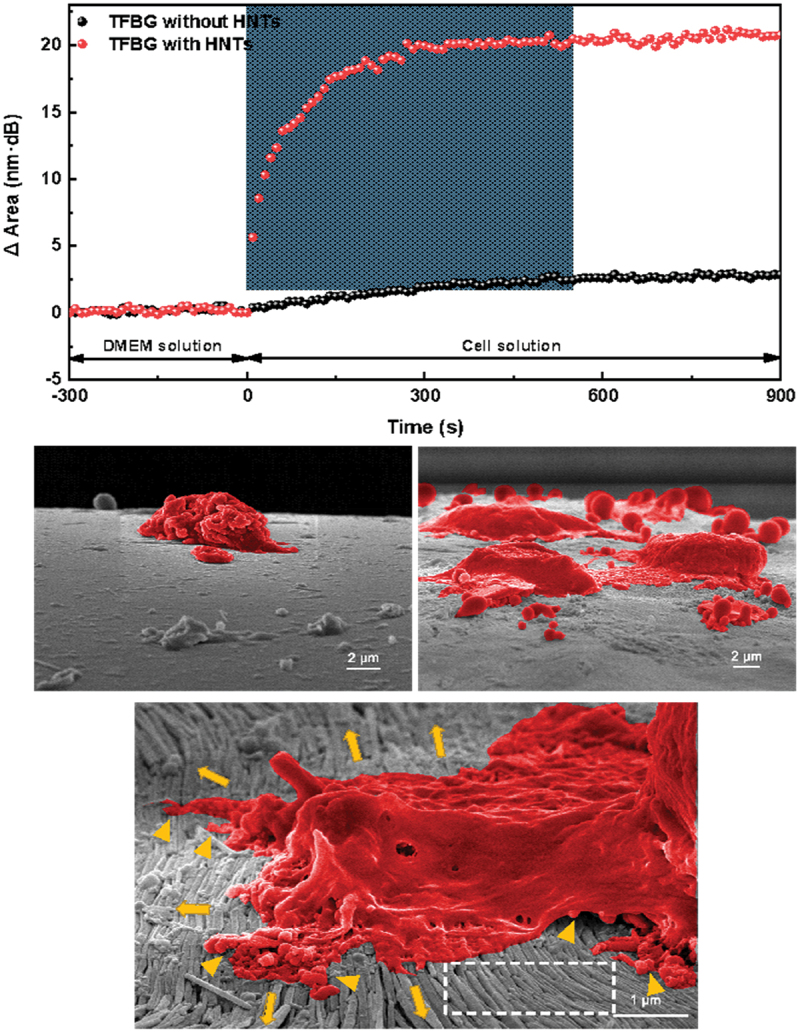
Real-time response of the spectral area of the tilted fiber Bragg grating substrate coated and uncoated with HNTs when MCF-7 cells were captured from the solution. Bottom: SEM images of uncoated substrate capturing the cells (left) and of HNT-coated support, which is more efficiently capturing MCF-7 (right), also with a zoomed view of one cell. Reproduced with permission from [26], copyright by Elsevier, 2023.

Apart from brush orientation of HNTs utilizing confined space, the nanotubes can be aligned into a strip-like pattern in confined spaces including glass capillary tubes, slit-like spaces, sphere- and pyramid-flat structures (Scheme 3(a–d)). For example, a confined space was formed between a glass or stainless-steel sphere and solid slides. The capillary and friction forces balance the HNT dispersion in the confined space, which leads to a periodic pinning and depinning deposition process. This gives the formation of a regular pattern on the substrates [21–25]. The HNTs pattern not only can guide the cell growth but also can capture tumor cells from physiological medium, Figure 3. The pattern was conjugated with anti-EpCAM, which leads to the capture yield of MCF-7 cells reaching 92% within 3 h. HNTs pattern can capture eight MCF-7 cells from 1 mL artificial blood samples with 10 cells, showing the promising applications in clinical tumor cell capture for early diagnosis and monitoring of cancer patients [26].

Recently, these clay nanotubes were arranged in an orderly manner along the optical fiber surface to form slit-like patterned layers to enhance the interaction with tumor cells, resulting in more effective capture of the cells in real time [26]. The sensor consists of a tilted fiber Bragg grating inscribed in the fiber core and specially designed HNTs coat. Such a sensor provides a powerful light-scattering sensing ability. Based on the spectral area interrogation method, normal and tumor cells can be unambiguously discriminated within a few minutes, providing high sensitivity (limit of detection of 10 cells/mL), and a linear response for a large cell concentration range (10^2^ ~ 10^5^ cells/mL). By integrating the fiber sensor with a microfluidic chip, rapid and precise measurement of tumor cell samples with low consumption (sub-microliter volumes) can be achieved.

## 3. Functionalization of biological microfibers with halloysite coating

Halloysite nanotubes were readily adsorbed onto biological microfibers, forming a 1–2-micrometer-thick coating on human or animal hair, wool, and cotton, and even on polyethylene terephthalate microfibers used for medical masks. Clay nanotubes are not oriented in such coating, but their thickness, density and stability may be adjusted with HNT dispersion concentration, pH, hydrophobicity, and by loading with functional chemical agents: dyes, medicines, or flame-retardants [27–30]. We assume that orientation is also possible along these microfibers, but it has not been demonstrated yet, probably due to relatively rough surface of such fibers. Clay nano-encapsulation of dyes allows avoiding a paradox in the process of hair coloring when one is trying to use an aqueous dye solution to achieve a not-water-soluble hair coloring. Encasing of dye or drugs into nanoclay containers allows for developing stable in water-based pigments (like color-loaded clay nanotubes or rain-resistant anti-lice clay formulation), which can then be assembled onto hair from aqueous dispersions. A similar approach was developed for halloysite modification of wool and cotton accomplished with stabilization through cross-linking of the nanotube-loaded alginate.

### 3.1. Halloysite hair coating (human and animals)

**Figure 4.**
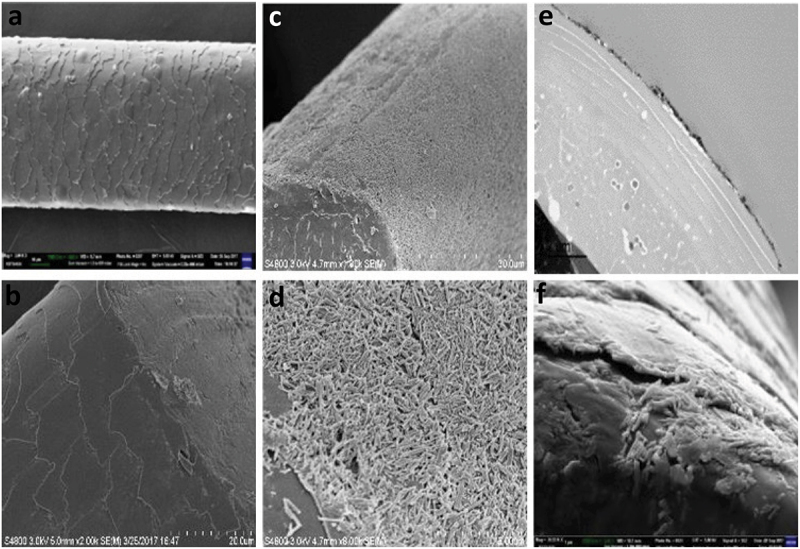
SEM images of pristine hair (a, b), halloysite-coated hair (c–f), side views and cross-sections. Reproduced with permission from [27], copyright by Royal Society of Chemistry, 2018.

**Figure 5.**
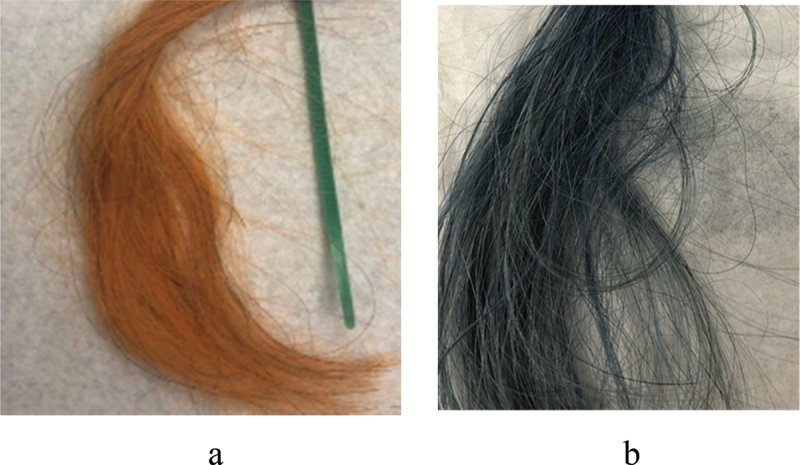
Grey hair coated with HNT-curcumin (a) and with HNT-indigo (b) formulations.

A spontaneous human hair coating with HNT was demonstrated when hairs were exposed for a few minutes to a 2–5 wt % aqueous dispersion of this nanoclay [27]. This finding attracted the interest of the cosmetic industry as a method for hair treatment and coloring with halloysite-loaded with medicine, like keratin or anti-lice drugs, or with a wide palette of dyes (Figures 4–5). One can load clay nanotubes with water-soluble dyes, especially efficient for anionic dyes like acid red or acid black, and then apply them to hair for coloring. Importantly, halloysite also allows to involve in such coloring poor water-soluble dyes, like natural brown lawsone or curcumin and blue indigo (Figure 5). Loading them from an acetone solution into the nanotubes with hydrophobized lumen and then safely applying such a nanoclay complex to human hair from an aqueous clay dispersion allows for a stable coloring [27]. One of the problems that has been overcome is color retention during multiple hair shampoo washing. Combining HNT self-assembly with preliminary hair treatment using selected polycations serving as a conditioner, one enhances electrostatic attraction with negative clay nanotubes and reached coated hair color stability close to the cosmetic industry standards.

**Figure 6.**
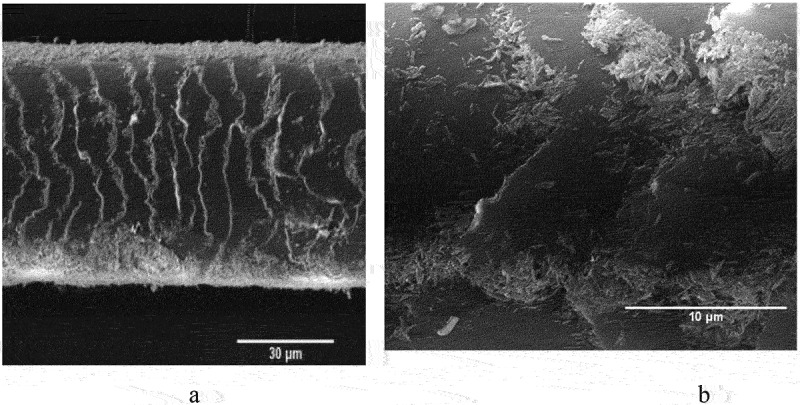
Halloysite coating process starts from cuticle edge area (after 2 and 3 min of deposition, a, b).

The study of HNT deposition kinetic has shown that in the first 1–2 min, the nanotubes were assembled in the vicinity of gaps of the hair surface scales, called cuticles, and then spread to a larger area (Figure 6). This phenomenon may be related to the non-even hair surface charge distribution. Also, hydrophobizing of halloysite enhanced hair coating. Related effect for enhanced halloysite coating was found for aquaria habitant animals, like capibara, whose fur contains ca 5 wt % of wax, which drastically improved the coating [28,29].

Sustained anti-lice animal fur protection was elaborated with a 1–2 µm coating layer of halloysite nanotubes via spontaneous self-assembly. A comparison of halloysite hair coating on aquatic and land-dwelling animals delineated the advantage of enhanced fur hydrophobicity with wax content. This finding was used to stabilize the clay nanotubes coverage for farm animals through pre-treatment with diluted wax. The coverage on such pre-treated animal hair/fur was protected for multiple washings, assuming stability against rain wettings [29]. The clay nanotubes were loaded at 6 wt % with an anti-lice permethrin and assembled on a goat fur, providing a slow drug release. The coating demonstrated efficient anti-lice protection for over one month, even after 2–3 washing cycles, thus withstanding rains, while the direct drug treatment from alcohol solution was terminated after the first washing. Such long-lasting topical hair/fur drug delivery may be applicable for other animals and human anti-lice treatments, as well as against fungal infections.

A very interesting medical application involves coating human hair coating with halloysite loaded with keratin allowing for UV protection [30]. The loaded clay nanotubes produce a micrometer-thick protective coating on the hair. Above the keratin isoelectric point (pH 4), negative protein adsorption into the positive halloysite lumen is favored, increasing zeta-potential magnitude to −43 mV and allowing for an enhanced colloidal stability of the formulation. Such 1 wt. % keratin-clay nanocomposite treatment demonstrated 60% of the hair surface coverage. This halloysite/keratin coating prevents the UV deterioration of the human hair as monitored by significant inhibition of cysteine acid.

### 3.2. Assembly on microfibers for blood clogging and flame-retardant enhancement

**Scheme 4.**
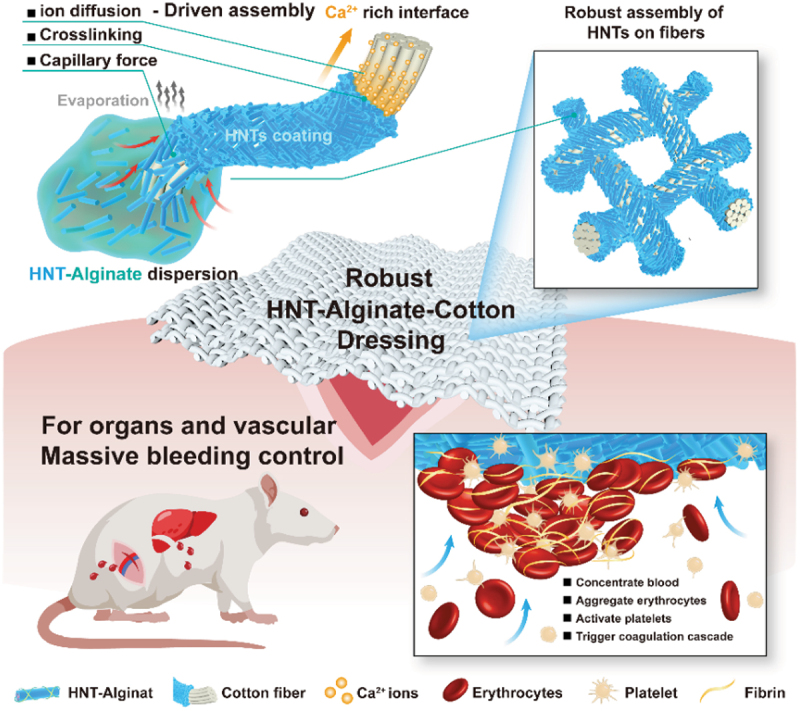
Schematic of the robust HNT assembly on cotton and an application in wound hemostats [32]. Reproduced with permission from [32], copyright by Wiley, 2023.

By an immersing and drying method with interfacial crosslinking, HNTs can be assembled on cotton and polymeric microfibers. This nanoclay coating could improve the hydrophilicity, flame-retardant property, separation performance, and blood clotting properties of the modified tissue. HNTs show negative a charge with high dispersion stability, which was exploited in the assembly and subsequent applications in functionalized microfiber tissue, Scheme 4.

The blood coagulation efficiency of HNTs lies between zeolite and montmorillonite, and it is 2.3 times faster than kaolin. Polyethylene terephthalate (PET) fiber dressing coated with HNTs was developed using a simple immerse-coating method [31]. Physicochemical characterizations such as XRD patterns and FTIR spectra of HNTs-PET suggest an anchoring of clay nanoparticles on the microfiber surfaces. The clotting time display that the coagulation capacity of PET fiber improves with the increase in HNTs content. The 2% HNTs treatment of PET was sufficient to achieve the hemostatic effect. FE-SEM and POM images suggest that the rod-shaped HNTs were tightly and evenly distributed on the PET fiber surface. HNTs bind on individual fiber rather than forming aggregates, and the introduction of HNTs does not cause fiber–fiber adhesion. TGA and DTG demonstrate that the HNT on dressing consists of 15–20 wt %, which allows for high hemostatic efficiency. The average blood loss with HNTs-PET was 0.41 ± 0.14 g, which is half of the blood loss of untreated PET dressing of 0.99 ± 0.14 g, demonstrating that the nanoclay coating is significantly better. The liver and femoral arterio-venous injury models also confirmed the fast hemostasis and reduced blood loss of the HNT-coated fibers. The adhesion evaluation showed that the nanoclay coating not only controls the sustained bleeding of epidermal wounds but also reduces the adhesion of the fibers to wounds. In addition to PET fibers, this technique was successfully applied to cotton [32].

To avoid the peel-off of HNTs from the polymer fibers, the clay was tightly bound onto commercial cotton fibers with alginate loaded in lumens and then finished with Ca^2+^ crosslinking [32]. This dressing materials maintain high procoagulant activity even after water treatment. Compared with commercial hemostats QuikClot® Cambat gauze, HNTs-alginate-cotton composite dressing exhibits hemostatic properties both in vivo and in vitro as well as high safety. The hemostats mechanism of the dressing was attributed to activating platelets, locally concentrating clotting components, and alginate cross-linked with Ca^2+^. The robust self-assembly of HNTs on textile fibers offers material with balanced high hemostatic activity, minimal ingredient loss, and biocompatibility. The clay-coated cotton dressing shows a great potential as hemostatic formulation for hospitals and home first aid kit.

The assembly of the clay nanotubes on polymer or cotton microfibers can also improve the flame-retardant properties. For instance, HNT coating can prevent polyurethane from burning and dripping. Also, the methylene blue adsorption capacity of HNTs-coated polymer foam increases from 0.02 to 0.15 mg g^−1^ and can reach 98% after 10 s. If HNTs are filled or modified with functional molecules (such as Ag or ZnO), the coated fibers will show antibacterial properties and become bioactive [32].

### 3.3. Nanoclay cotton fiber coating using layer-by-layer self-assembly

**Figure 7.**
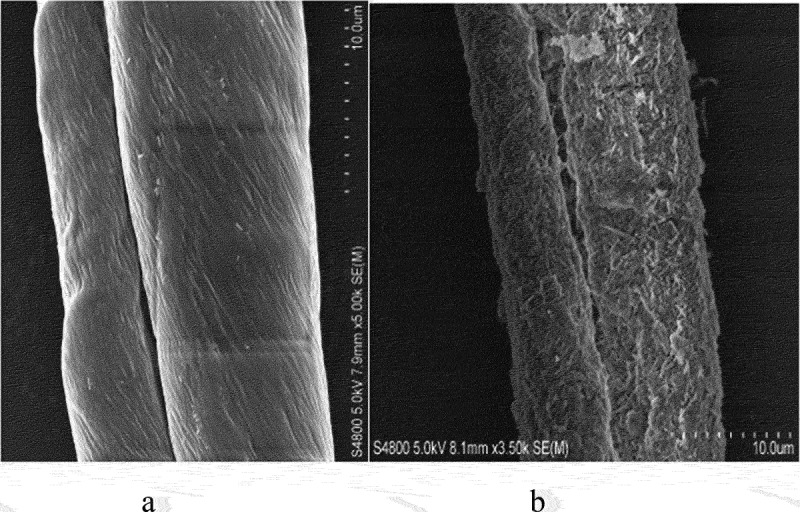
SEM images of the pristine uncoated cotton fiber (a) and the fiber with HNT + PEI/HNT multilayer coating (b). Reproduced with permission from [34], copyright by ACES, Asian Chemical Editorial Society, 2023.

**Scheme 5.**
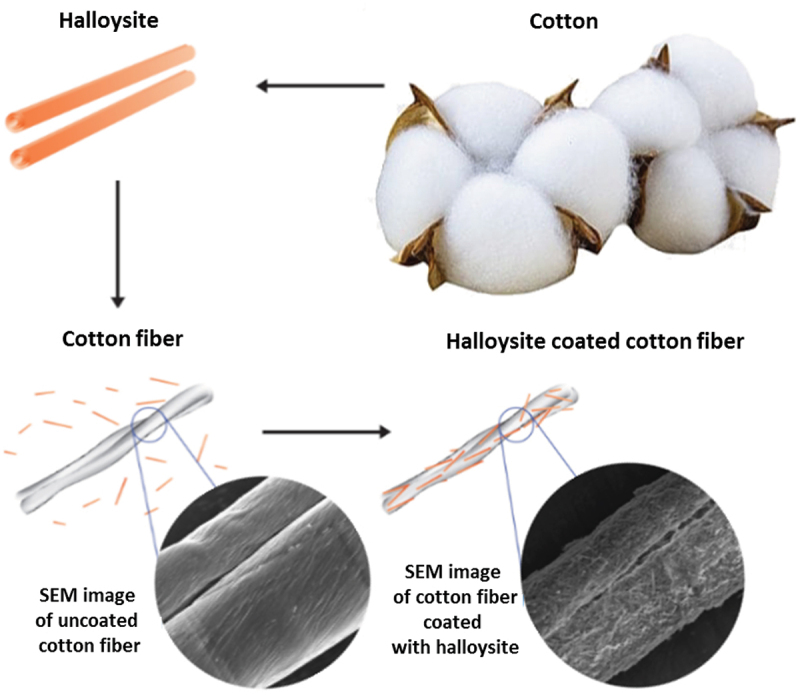
Alternating coating of cotton with PEI/HNT, and SEM images of untreated and treated cotton microfibers.

Even more efficient was HNT coating combined with layer-by-layer (LbL) method providing polycation underlayer, which then alternate with anionic clay nanotubes, allowing an optimal clay cover thickness. This may give modification of fabric, providing it with new colors, and enhanced flame retardancy (Scheme 5) [33,34]. Among the most often used fabrics are cotton and its assortments. Cotton is a very flammable substance, and a retardant coating can lessen its fire danger. Using Louisiana-grown raw cotton, we developed an architectural organic/inorganic fiber coating using LbL assembly of the anionic HNT alternated with cationic polyethyleneimine (PEI). Upon burning the cotton fibers covered with the nanoclay to assess the level of fire safety, we found that the flame spontaneously extinguished. The optimum level of flame retardancy for such treated cotton was found to be achieved by adding two bilayers of PEI/HNT, which made up 7 wt % of the tissue, Figure 7. It is not practical increasing the coating thickness because the flame retardancy increased at a lower rate with additional layers. Furthermore, the clay nanotubes were also loaded with color-boosting dyes and antimicrobials, giving the coated cotton tissue a better look and functionality. This nano-compositional textile product presents a promising industrial technology combining abundantly available cotton and clay, both are natural products.

The coating on the cotton was first done by HNT adsorption from its aqueous dispersion, which is stabilized using PEI layer, acting essentially as a cationic glue. Further, alternate layers of HNT and PEI were added until the desired coating architecture was reached. From the thermo-gravimetric (TGA) analyses, it was observed that the first layer added of about 5 wt % of the nanoclay, whereas the subsequent addition of layers gave about 2 wt %. The coating thickness for one bilayer may be estimated to be about 0.4 ± 0.1 μm, with halloysite density of 2.54 g/cm^3^ being twice as large as cotton. The cotton strips were subjected to a vertical flame test, and the speed of burning and the length of the fiber burned were recorded. The results showed that with the increasing layer numbers, the flame retardancy was enhanced, albeit at a diminishing rate. While the cotton with just the first layer of HNT burned the entire length under 10 s, the cotton with two clay bilayers burned slower and only partially, with about one-third of the total length. It was observed that the cotton with higher number of bilayers showed diminished effect on the self-extinguishing abilities. Thus, we regard two bilayer HNT/PEI coating to be optimal for cotton flame retardancy.

In addition to flame retardancy, we showed that cotton can be functionalized with HNT loaded with antibiotics and dyes, showing that we can expand this technology to produce colored textile coating with antibacterial protection. Antibacterial activity of HNT coated cotton was evaluated with a chloramphenicol–nanoclay formulation on *Serratia marcescens* microbes grown on nutrient media. The results showed that the inhibition zones for cotton treated with PEI+HNT-chloramphenicol were bigger compared to just PEI with chloramphenicol, thus demonstrating an effective antibacterial coating on the colored tissue [34]. The suggested nano-architectural design on microfibers shows a promising technology where antibacterial and dye agents can be encapsulated in HNT assembled on cotton for sport, home, and car textiles. Such textiles coated with loaded nanoclay are safe because only biocompatible HNT encounters the skin, providing human friendly environment.

## 4. Pickering halloysite-oil emulsion and reversed HNT-marbles for cell encasing

**Figure 8.**
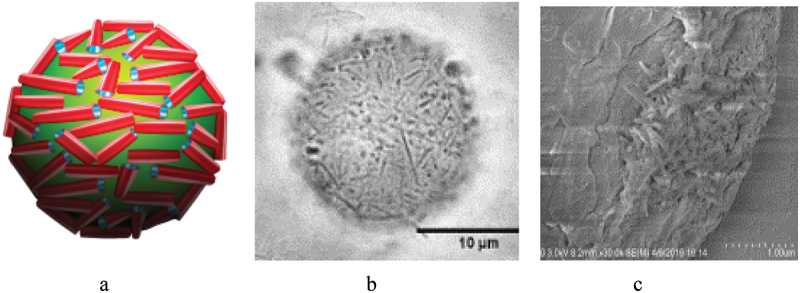
Scheme of HNT location on oil microbubble (a), and experimental optical (b) and cryo-SEM images (c) of petroleum droplet coated with halloysite. Reproduced with permission from [38], copyright Elsevier 2018.

Aqueous clays exhibit amphiphilic properties and may emulsify oil, which is especially efficient for easily dispersible halloysite nanoclay. The addition of 1 wt % aqueous halloysite to 30 wt % of hexadecane in water, representing a model petroleum, allows to disperse it after short stirring [35–38]. Hydrophobizing these clay nanotubes with ODTM salinization or with carbonized chitosan coating, giving for water contact angle close to 90º, allowed for stable 5–10 μm droplet diameter oil emulsification even in sea water at 5 M NaCl. Halloysite nanotubes form a 0.5 μm thick shell around the oil droplet, minimizing water–oil interfacial energy (Figure 8). This interfacial halloysite layer surprisingly resembled the hair coating in Figure 4. The method also worked for crude petroleum emulsification, such as Macondo Louisiana type, thus promising a technique for spill oil remediation. An additional advantage of this approach is the exploitation of the biocompatibility of halloysite nanotubes, which, contrary to commonly used Corexit® detergent composite, do not depress proliferation oil-eating marine microbes, such as *Alcanivorax borkumensis* [38].

**Figure 9.**
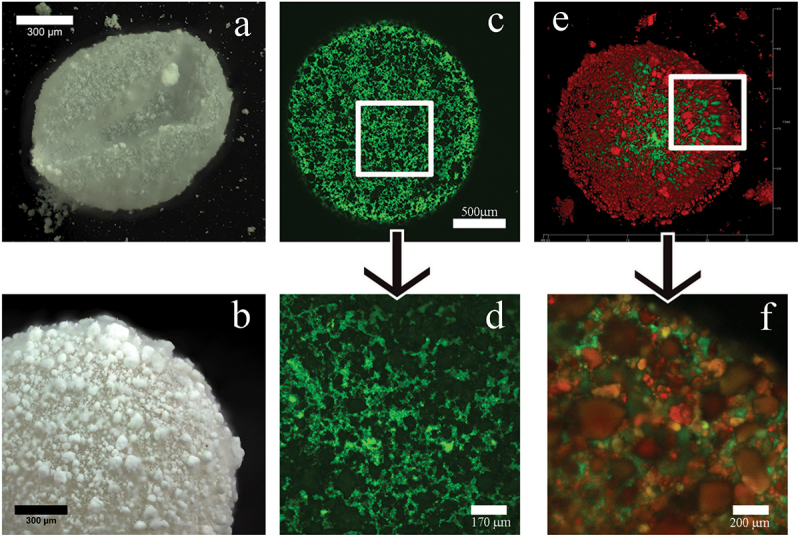
Dark-field microscopy images of HNT marbles (a, b). Confocal microphotographs of liquid marbles and its surface. Cells A. *Borkumensis* are stained by vital dye dihexyloxacarbocyanine iodine – DioC_6_ inside the marble (c, d). Images of liquid marbles with halloysite nanotubes, stained by rhodamine (red) and *E. coli* are stained by green DiOC_6_ (e, f). Reproduced with permission from [39], copyright by American Chemical Society, 2019.

The above section describes the formation of a Pickering emulsion based on hydrophobic oil droplets stabilized with interfacial halloysite in hydrophilic water media. The reverse situation is also possible, when inner water droplets are stabilized by halloysite at the interface with hydrophobic air [39]. Dark field optical and laser scanning confocal microscopy of the liquid marbles confirmed this formation. It involves a simple rolling of a water droplet on some powder of ODTMS-treated HNT, allowing for observing the structure composed from red-stained halloysite and green-colored bacterial biofilm (Figure 9). First, we showed the overall 3D structure of the cell-filled marbles in the air to confirm the flexible behavior of halloysite-built shells. Further, fluorescence confocal images demonstrate the viability of the encapsulated cells and expose the mutual distribution of red dye-labeled halloysite and green bacteria. Such halloysite encapsulation allowed preserving bacteria at room temperature without refrigerating for 2 weeks.

## 5. Core–shell halloysite-based nanosystems

**Scheme 6.**
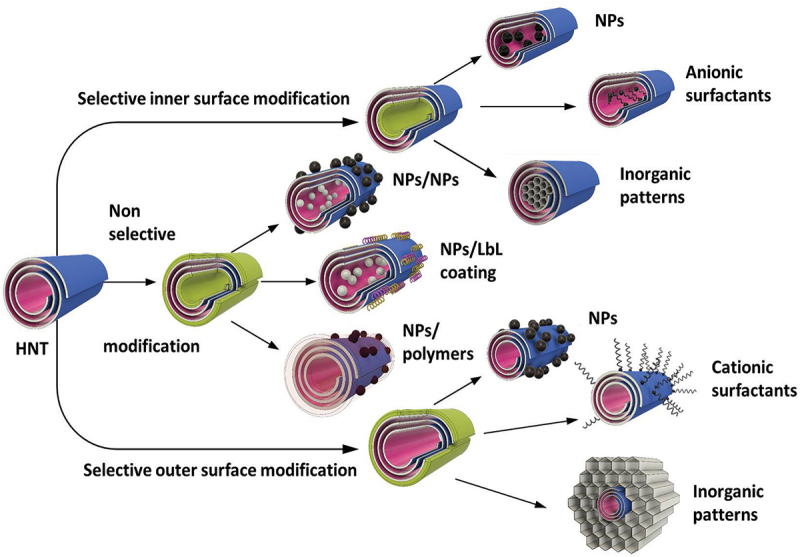
Different architectures of halloysite nanotube-based core–shell systems.

Numerous biomedical research have been conducted with nanosystem where inorganic and organic blocks were placed on halloysite in desired positions. This became possible due to nano-architectural approach in the design of hybrid core–shell structures [8,12]. Developed methods have shown ample opportunities for the production of ceramic, metal, and semiconductive nanoparticles with core–shell tubule systems with selectively modified inner or outer surfaces of halloysite (Scheme 6).

One of the unique perspectives is the modification of the inner surface with catalytically active nanoparticles. The inner tubes modification using surfactants or chelating agents followed by particles formation results in nanoreactors with enhanced catalytic properties. In [40], the advantages of halloysite-based nanoreactors for petrochemical processes are described. RuCo and Ru nanoparticles, selectively produced inside the clay nanotubes using ligand-assisted modification, showed the process selectivity in Fischer-Tropsch synthesis. Applications of urea, azines, or EDTA (ethylenediaminetetraacetic acid) resulted in different acidity of materials, and as a result, different selectivity for liquid hydrocarbons and waxes.

Modification of the outer surface of halloysite with cetyltrimethylammonium bromide – CTAB and further sol-gel formation of silica/aluminosilicate composite (Mobil Composition of Matter, MCM-41), with halloysite intercalation, was used to develop new catalytic nanocarriers with enhanced thermo- and mechanical stability. These composites can be used for the removal of pollutants and heavy metals from wastewater due to their high adsorption capacity and selectivity. The porous nature of MCM-41-halloysite composites makes them suitable for gas storage applications, such as hydrogen storage, which is important for clean energy technologies. These composites can be employed in the development of sensing devices for detecting gases, chemicals, or biomolecules, thanks to their high surface area and ability to interact with analytes.

**Figure 10.**
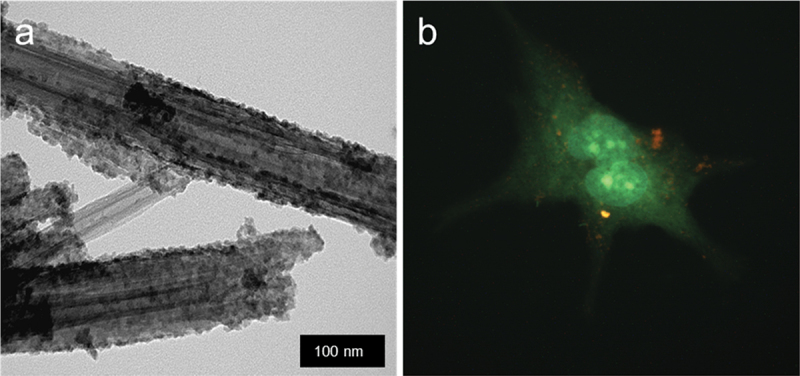
CdS nanoparticles on halloysite tube (a) and fibroblast cell labeled with these bright core–shell quantum dots (b) reproduced from [42], an open access publication by MDPI (Multidisciplinary Digital Publishing Institute), 2018.

Synthesis of quantum dots (QD) on halloysite for biomedical application was suggested [41,42]. To stabilize these dots on the surface of clay, nanotubes grafting agents like silanes and azine were used. Depending on the QD composition (CdS, ZnS, CdZnS) and grafting agent, these nanoparticles demonstrate different light adsorption, fluorescence, and band gap energy. The use of halloysite as a template allowed for the synthesis of almost monodispersed QDs with a size of 6–7 nm through a simple route in an ethanol solution using co-precipitation of Zn, Cd and Zn nitrates with thioacetamide (Figure 10). Nanoarchitectural optimization helped with the nanodots’ tunable properties, as was tested for stable fluorescent labels in bioimaging of cancer cells (Figure 10(b)). These nanomaterials possess no acute toxic effects on the model nematodes and have a high viability in tests with mammalian cells. Such materials could be used for target cancer therapy with monitoring the process online due to their light-responsive properties. 27-nm gold clusters also showed stable fluorescent signals after grafting to halloysite nanotubes [43]. Biocompatibility of the resulted Au-HNT formulations minimizes the risk of adverse reactions or toxicity. By functionalizing the nanotubes with specific ligands or antibodies, one may direct them to specific cells or tissues, increasing the effectiveness of the treatment. Adsorption of Ag and Au on HNT increases the local plasmon resonance, which is perspective for further investigated. Such nanotube-grafted Ag and Au nanoparticles also showed good performance in spatially localized hyperthermia [41].

## 6. Halloysite biosafety

Halloysite, either as a component of polymer-based composites, or as a stand-alone material (e.g. a platform for drug delivery), has been extensively studied for its biocompatibility and toxicity. Most toxicity investigations are performed in vitro and rarely in vivo. The toxicity of halloysite-based materials may be caused not only by the clay itself but also by the other components; therefore, one must differentiate between pristine and modified halloysite [44]. It appears that halloysite does not pose any significant toxicity both in vitro and in vivo, with concentrations up to 0.5 mg/mL, but this depends on the administration pathway. Halloysite is a natural material, which implies that its properties may differ depending on the origin, pre-treatment, impurities, and individual particles morphology. Therefore, it is important to compare halloysite obtained from different sources in a single experimental setup.

Cytotoxicity and genotoxicity of different halloysites were investigated in vitro employing several mammalian cell lines (CHO, HeLa, and HepG2) and results confirmed their exceptional biocompatibility, independently of origin. However, if the concentration was increased above 0.6 mg/mL, moderate toxicity was observed [45].

The effects of halloysite on cells in culture were evaluated in comparison with multiwall carbon nanotubes [46]. Both types of nanotubes were assayed after administration to human umbilical vein endothelial cells and mice (blood vessels were studied). The results suggested that halloysite was internalized by these cells via a non-endocytic pathway. Both halloysite and carbon nanotubes induced autophagy dysfunction, which activated the apoptosis and caused the mitochondria dysfunction. Carbon nanotubes induced significantly higher pronounced inhibitive effects, while halloysite toxicity was much less. HUVECs and MCF-7 human cell cultures were also studied simultaneously in in vitro model, using the identical halloysite specimens. The results indicated a very low cytotoxicity up to 0.5 mg/mL.

The acute toxicity of halloysite clay nanotubes in vivo was analysed employing the nematode *Caenorhabditis elegans* as a model organism. Halloysite was localized exclusively in the alimentary system, and it does not induce severe toxic effects on nematodes within the concentrations up to 1 mg/mL [47]. The nanosafety of halloysite was also confirmed employing fish (*Danio rerio*) as an in vivo model [48]. In zebrafish, the safe halloysite concentration was much higher than in cell experiments, reaching 20 mg/mL, and no acute toxic and or teratogenic effects were observed. The authors noticed that halloysite dose-dependently promoted hatching in zebrafish. Although halloysite itself and several HNT-based composites do not inflict significant toxicity or pose danger for living species, it is clear that effects of these inorganic nanotubes on biosystems, especially on the organism level, need to be studied further.

## 7. Conclusions

### 7.1. Interfacial coating

We have generalized results on have interfacial self-assembly of halloysite clay nanotubes, which allows for an architectural design of functional systems. These hydrophilic and negatively charged tubes, with a diameter of 50 nm and a length of approximately 1 μm, are spontaneously adsorbed from aqueous dispersions on biological microfibers and oil/petroleum microbubbles. Therefore, halloysite is capable of stabilizing at the border with water and both polar and nonpolar surfaces. To minimize interfacial energy for oil/water or water/air borders, one needs to increase with silanization the halloysite surface contact angle from pristine 20 to 90º. Surprisingly, such additional hydrophobization promotes clay nanotube assembly on hair. It may be related with nonuniform, patchy surfaces, containing charged and non-polar microdomains. Typically, halloysite interfacial formations include few lateral nanotube layers, and this coating thickness is of 0.5–1 μm. In case of hair and cotton, the assembly survives 7–8 shampoo washing. The coating may additionally be strengthened with some binding, for example, through co-loaded alginate linked through Ca^2+^ or with polycation pretreatment enhancing an electrostatic interaction with the nanoclay.

### 7.2. Surface micropatterning

The nanotubes were found to orient along the edge of a drying droplet of a halloysite suspension following the phenomenon known as ‘coffee ring’ formation. During drying, the three-phase contact line among the air, suspension, and solid substrate remains fixed, and evaporation pulls the fluid from the center of the droplet, pushing the suspended nanoparticles toward the periphery. The accumulation of tubes from the center to the circumference increases the tubes’ concentration beyond a critical level and causes their orientation. Better orientation may be reached with enhancing the nanotube electrical zeta-potential from original −30 to −60 mV. The crowding at the contact line converts the halloysite into the nematic phase, aligning the nanotubes. In capillaries vertically placed in an hot oven for accelerated drying, the contact line falls to a lower position, forming periodical rings of oriented nanotubes. Manipulation with contact angle breaks during the water drying allows one to create patterns. Such patterns may be regular inner rings inside the capillary, lines on two parallel flat glasses with halloysite dispersion placed in between when dried at 60–70ºC, or concentric circles with the nanoclay colloid dropped on tiny balls on the substrate and dried. Brash shear-force halloysite deposition gives a similar effect at larger surface area. Selective tumor cell capturing and stem cell differentiation were observed on aligned clay nanotube patterns.

### 7.3. Functionalizing through the tube loading

Halloysite tubule nature allows for its loading with active chemical agents, dyes, and drugs, providing an additional functionality for the coating, such as color, antibacterial, cell differentiation or specific adsorption, and fiber flame-retardancy. This chemical load may usually be released in water in 30–50 h, and can have an extended release, if clogged with polymers. One could make the halloysite cargo permanent, like for coloring, using loading with water insoluble dyes from acetone. The nanotube load is restricted inside, allowing for decreased toxicity of cadmium quantum dots, making them convenient for biological labeling or for cosmetics, assuming that only safe clay surface comes in contact with skin. Suggested halloysite nanotubes are biocompatible and alive organisms’ friendly material, which fits well for many biomedical applications, including topical drug delivery.
